# Single Versus Double Implants in a Narrowed Mandibular Molar Space: A Finite Element Analysis

**DOI:** 10.3390/jfb17100533

**Published:** 2026-10-21

**Authors:** Elif Aktürk, Melis Banri, Gülsüm Sayın Özel

**Affiliations:** 1 Department of Prosthodontics, Institute of Health Sciences, Istanbul Medipol University, Beykoz, 34815 Istanbul, Türkiye; 2 Department of Prosthodontics, Faculty of Dentistry, Istanbul Medipol University, Beykoz, 34815 Istanbul, Türkiye

**Keywords:** dental implants, single-tooth, dental prosthesis, implant-supported, alveolar bone loss, finite element analysis, dental stress analysis

## Abstract

After molar loss, tipping of the adjacent teeth and extrusion of the opposing tooth often leave a mesiodistal space too narrow for a conventional implant. This study compared stress in the implant components and peri-implant bone among single- and double-implant configurations of different diameters in such a space. A mandibular model was constructed by computer-aided design; ridge resorption and mesial tipping of the second molar left a 13.6 mm mesiodistal space and a 7.5 mm crestal ridge width. Four configurations were modeled: 4.8 mm single (M1), 4.1 mm single (M2), 3.3 + 3.3 mm double (M3), and 4.1 + 3.3 mm double (M4). Vertical (150 N) and oblique (300 N at 45°) loads were applied; von Mises stress in the components and maximum and minimum principal stresses in bone were recorded. Oblique loading raised stress in every structure; because the oblique load was twice the vertical load, part of this increase reflects the higher force rather than loading direction alone, and direction-related increases (normalized per unit load) ranged from approximately 2- to 9-fold across components and bone. Under vertical loading, M1 gave the lowest implant body, abutment, and screw stresses (31.0, 36.7, and 16.1 MPa). Under oblique loading, M4 lowered implant body and abutment stress, whereas M1 retained the lowest screw (192.0 MPa) and configuration-level bone stresses (31.2 and 7.2 MPa). Because screw preload was not modeled, screw-stress comparisons should be interpreted as relative mechanical demand rather than as predictors of loosening or fracture risk. No configuration minimized stress in every structure. Implant diameter, site, component, and loading condition should be weighed together.

## 1. Introduction

The rehabilitation of complete or partial tooth loss with dental implants improves masticatory efficiency, patient satisfaction, and overall quality of life [1]. The mandibular first molar is among the most frequently restored teeth in implant dentistry [2]. Following tooth extraction in this region, tipping of adjacent teeth, supereruption of opposing teeth, and alveolar ridge resorption may progressively reduce the available mesiodistal space [3,4]. During implant placement in the posterior region, a minimum distance of 1.5 mm between an implant and an adjacent tooth and 3 mm between two implants is generally recommended to preserve peri-implant tissues [4,5]. Failure to maintain these distances may compromise biological and prosthetic outcomes. Furthermore, implant placement in anatomically narrowed spaces may necessitate unfavorable positioning, potentially increasing stress concentration at the bone–implant interface under nonaxial loading [3,6]. Excessive stress in the peri-implant region may contribute to marginal bone loss over time [7].

In narrowed posterior spaces, the selection of an appropriate implant configuration depends primarily on the available mesiodistal space and buccolingual bone width [2,8]. When the bone width is sufficient and the mesiodistal space is limited, a single wide-diameter implant may be preferred because of its simpler restorative design and easier maintenance of oral hygiene [2,8]. Conversely, when the mesiodistal space is wider, restoration with a large crown supported by a single implant may result in unfavorable force distribution and increased bending moments. In such situations, placement of two implants has been proposed as an alternative treatment approach [9].

Previous studies have reported conflicting findings regarding the biomechanical advantages of single- and double-implant configurations. Wide-diameter implants have been associated with more favorable stress distribution, whereas narrow-diameter implants may exhibit less favorable biomechanical behavior, particularly under loading conditions [10]. Nevertheless, when the available bone width is insufficient for a standard-diameter implant and grafting procedures are not feasible, two narrow-diameter implants may represent an alternative treatment option [11,12,13]; clinical syntheses indicate high survival of narrow-diameter implants supporting fixed restorations in the posterior region [13]. Splinting two implants has been reported to provide more favorable stress distribution in the surrounding bone, reduce the unsupported prosthetic span, and decrease the risk of screw loosening compared with a single-implant configuration [9,14,15]. Conversely, de Carvalho et al. [16], using strain-gauge analysis, reported lower peri-implant strain around a single implant than around two implants. Clinical studies have also reported mechanical and biological complications associated with single-implant molar replacement, including prosthetic mobility and implant loss [17,18,19]. These conflicting findings indicate that the biomechanical advantages of single- versus double-implant configurations remain unclear, particularly when implant diameter and available anatomical space are considered simultaneously.

The biomechanical evaluation of stress distribution in both peri-implant bone and implant components is critical for the long-term success of implant-supported restorations. Excessive peri-implant stress may contribute to microdamage, marginal bone loss, and deterioration of implant support, whereas stress concentrations within implant components may increase the risk of mechanical complications such as screw loosening, screw fracture, and, in rare cases, implant fracture [20,21]. Biomechanical studies have demonstrated that occlusal forces may concentrate particularly at the abutment screw and implant–abutment connection [22,23,24,25]. Furthermore, implant-related factors, including implant diameter, neck design, and connection type, can influence stress distribution within the implant–abutment complex [24,26,27,28]. Therefore, simultaneous evaluation of stress distribution in the implant body, abutment, screw, and surrounding bone is important for understanding the biomechanical behavior of different implant configurations.

Finite element analysis (FEA) is widely used in implant dentistry to evaluate stress distribution within implant components and surrounding bone under controlled loading conditions [22,29]. Previous FEA studies have compared single- and double-implant configurations for molar replacement [14,26,30] and the effect of implant number and location in other posterior spans [31]; however, these studies have generally modeled a single missing molar in bone with conventional dimensions. Limited evidence is available regarding the biomechanical behavior of single- and double-implant configurations with different implant diameters specifically in an anatomically narrowed, two-tooth mesiodistal space restored with a screw-retained titanium-base (Ti-base)/monolithic zirconia hybrid restoration, particularly when stress distribution is simultaneously evaluated at the component level in the implant body, abutment, screw, and peri-implant cortical and trabecular bone. The present model therefore extends previous comparisons by combining a narrowed and resorbed two-tooth span, a hybrid abutment–restoration design, and simultaneous component-level evaluation of the implant body, abutment, and screw. Therefore, the aim of this study was to compare stress distributions in the implant body, abutment, screw, and surrounding cortical and trabecular bone among single- and double-implant configurations with different implant diameters placed in a narrowed and resorbed mandibular molar region under vertical and oblique loading conditions using FEA. The null hypothesis was that implant number and diameter would not affect stress distribution in the implant components or surrounding bone under the evaluated loading conditions.

## 2. Materials and Methods

This FEA study compared stress distributions among two single-implant and two double-implant configurations with different implant diameters in a narrowed mandibular molar space. The three-dimensional bone geometry used in the models was obtained from the Visible Human Project dataset (National Library of Medicine, Bethesda, MD, USA). Because the study was performed entirely on computer-generated models derived from this publicly available anatomical dataset and did not involve human participants, human tissue, or identifiable patient data, institutional review board approval was not required.

A three-dimensional mandibular model encompassing the first premolar to second molar region was constructed in a computer-aided design (CAD) environment according to the dimensions defined for this study. The cortical shell was initially modeled with a uniform thickness of 2 mm, and the trabecular bone was generated from its inner surfaces. The teeth were modeled according to Wheeler’s atlas of tooth form [32], and the periodontal ligament was created by offsetting the outer root surface by 0.25 mm. Geometry preparation and reverse engineering were performed in ANSYS SpaceClaim (version 22.0; Ansys Inc., Canonsburg, PA, USA). All models were assembled and meshed in ANSYS Workbench (version 22.0; Ansys Inc., Canonsburg, PA, USA) and solved using the implicit solver of LS-DYNA (LSTC, Livermore, CA, USA). A mesh convergence test was performed on the M1 model (selected as representative) to ensure that the numerical results were independent of mesh density. Local mesh sizes of 0.4, 0.3, 0.2, and 0.1 mm, applied in the critical regions, such as the areas around the implant holes (and not as a global element size over the whole mandibular segment), were evaluated under identical loading and boundary conditions, using both the maximum von Mises stress in the implant and the maximum principal stress in the peri-implant bone under oblique loading as convergence criteria. For the implant-body stress, the relative differences between successive mesh refinements were 10.29%, 6.35%, and 2.21%, respectively. For the peri-implant bone stress, the values were 25.5, 28.7, 30.5, and 31.2 MPa at the four mesh sizes, with corresponding relative differences of 11.26%, 5.93%, and 2.29%. Because the relative error between the 0.2- and 0.1-mm meshes was below the predefined 3% convergence threshold, the 0.1-mm local mesh size was considered sufficiently converged and was used in the critical regions for the final analyses, whereas larger elements were used in the less critical regions of the model, such as the fixation areas. The models were meshed with linear (first-order), four-node tetrahedral solid elements (CTETRA4); triangular two-dimensional surface elements were additionally used to better capture the curved, organic geometry of the bone. Mesh quality was assessed against predefined criteria (element skewness ≤ 80° and minimum edge length ≥ 0.001 mm), and elements that did not meet these criteria were corrected before the final analyses. The total number of elements and nodes in the finalized models was 1,598,203 and 409,803 for M1; 1,458,659 and 375,372 for M2; 1,930,024 and 495,836 for M3; and 2,108,003 and 538,256 for M4, respectively. The element and node counts of every component of each model and the complete convergence data are provided in Supplementary Tables S1 and S2. Boundary conditions were defined by restricting displacement along the three spatial axes at the anterior, posterior, and inferior surfaces of the model.

To simulate a narrowed molar space, the second molar was tipped mesially, which reduced the mesiodistal contact-to-contact distance between the adjacent teeth from 18 mm (the combined mesiodistal width of the missing second premolar and first molar) to 13.6 mm, as occurs clinically after tooth loss, and the ridge at the edentulous site was narrowed to a crestal buccolingual width of 7.5 mm (from 11 mm), together with approximately 1 mm of vertical crestal bone loss. No separate mesiodistal bone reduction was applied. As part of the simulated resorption, the cortical shell was thinned by 1.0 mm on the buccal and 0.7 mm on the lingual aspect of the ridge, resulting in a crestal cortical thickness of approximately 1.0 mm buccally and 1.3 mm lingually, and the remaining reduction in the crestal width was taken from the trabecular core, thereby preserving cortical–trabecular continuity in the reduced region. The modeled crestal narrowing (3.5 mm, 32%) is within the range of horizontal ridge reduction reported within 6–7 months after tooth extraction (mean 3.79 mm; 29–63%), and the modeled vertical crestal loss (approximately 1 mm) is within the reported mean vertical reduction of 0.8–1.2 mm [33]. The original ridge width of 11 mm, as measured on the model, is close to the mean width reported for dentate mandibular first molar sites (10.2 mm [34]), and the resorbed crestal width of 7.5 mm is within one standard deviation of the mean width of edentulous first molar sites (6.5 ± 1.8 mm [35]). In double-implant configurations, the distances measured on the model from the neck of each implant at the level of the implant platform, which for the bone-level implants used corresponds to the alveolar crest, were 1.7 mm between each implant and the adjacent tooth and 3.4 mm between the two implants in M3, and 1.5 and 3.0 mm, respectively, in M4; in both models the implants and the gaps together spanned 13.4 mm at platform level (compared with the 13.6-mm contact-to-contact distance at crown level), and both models therefore satisfied the minimum 1.5-3-1.5-mm (6.0-mm) distances cited in Section 1. The first premolar was positioned vertically, whereas the second molar was modeled with a 29.1° mesial tipping angle, measured directly from the three-dimensional model and configured to represent a typical clinical presentation of ridge narrowing. The 29.1° mesial tipping is within the range reported for mandibular second molars after first molar loss (mean mesiodistal angulation 57.5 ± 10.3° versus 82.8 ± 5.1° in controls) [36].

Four implant configurations were evaluated: a 4.8 × 10-mm single implant (M1), a 4.1 × 10-mm single implant (M2), two 3.3 × 10-mm implants (M3), and a 4.1 × 10-mm molar implant combined with a 3.3 × 10-mm premolar implant (M4). The implants were modeled in a three-dimensional CAD environment according to the manufacturer’s specifications (Institut Straumann AG, Basel, Switzerland). Thread features of the implant body and the abutment screw were included in the models. All implants were Straumann Bone Level (BL) implants [37]. The 4.8-mm and 4.1-mm single implants (M1, M2) were restored using a hexed Regular CrossFit (RC) Variobase for Crown Ti-base (ref. 025.4921) with its corresponding screw (ref. 025.4900). The 3.3-mm implants in the double-implant configurations (M3 and M4) used a non-hexed Narrow CrossFit (NC) Variobase for Bridge/Bar Cylindrical Ti-base (ref. 022.0110) with a screw (ref. 025.2926), consistent with their splinted restoration; the 4.1-mm implant in the mixed configuration (M4) used a non-hexed Regular CrossFit (RC) Variobase for Bridge/Bar Cylindrical Ti-base (ref. 022.0111) with the same screw (ref. 025.2926). All Ti-base abutments had a 4.5-mm abutment diameter, a 3.5-mm abutment height, and a 1.0-mm gingival height; the restoration had a height of 8.4 mm from the ridge crest to the cusp tip, measured directly from the three-dimensional model. In the single-implant models, the implant was positioned centrally in the region of the first molar, and the crown was modeled to fill the entire mesiodistal space between the adjacent teeth, which results in a mesiodistal overhang of the crown relative to the implant platform. In the double-implant models, the implants were positioned according to the anatomical molar and premolar regions, considering the crestal bone level, adjacent tooth roots, and available geometry of the narrowed space.

**Figure 1:**
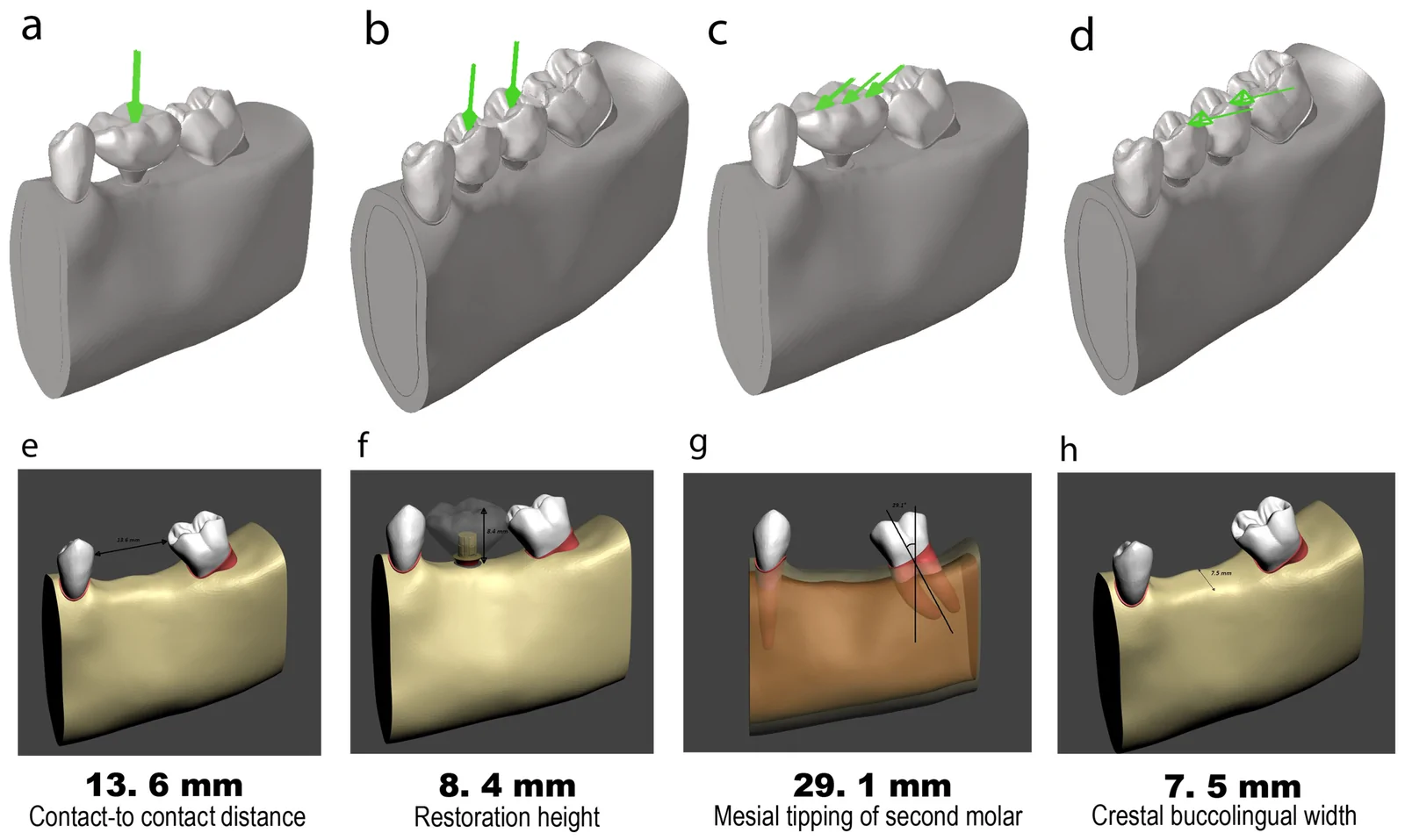
Loading conditions (**a**–**d**) and model geometry (**e**–**h**) used in the finite element analysis. (**a**) Vertical loading of the single-implant model; (**b**) vertical loading of the double-implant model; (**c**) oblique loading of the single-implant model; (**d**) oblique loading of the double-implant model. For vertical loading, a total force of 150 N was applied to the central fossa parallel to the long axis of the implants. For oblique loading, a total force of 300 N was applied to the lingual inclines of the buccal cusps at a 45° angle to the long axis of the implants. In the double-implant models, the total load was divided equally between the two restorations. Panels (**e**–**h**) show the narrowed molar space as modeled and the dimensions measured directly on the three-dimensional model: (**e**) mesiodistal contact-to-contact distance, 13.6 mm; (**f**) height of the restoration from the ridge crest to the cusp tip, 8.4 mm; (**g**) mesial tipping of the second molar, 29.1°; and (**h**) buccolingual width of the ridge at the crest, 7.5 mm.

In all models, complete osseointegration was assumed, and the bone–implant interface was defined as fully bonded. Screw-retained monolithic zirconia restorations were supported by blum-base (Ti-base) abutments; in the double-implant configurations (M3 and M4), the two abutments were restored with a single, one-piece (splinted) monolithic zirconia restoration consisting of two premolar-shaped units, rather than two separate crowns. A uniform 30-µm self-adhesive resin cement layer was modeled between the zirconia restoration and the Ti-base, with bonded contact assumed at the zirconia–cement and cement–Ti-base interfaces. The Ti-base abutments were connected to the implants through a screw-retained connection, the precision of which can influence the quality of the implant–abutment interface [38]. Bonded contact was defined between all components of the finite element models, assuming no relative displacement at the interfaces. The effect of screw preload was neglected in the analyses; the study aimed at a relative comparison among configurations under identical, simplified (fully bonded) conditions, and this simplification is discussed further as a limitation below. For vertical loading, a total force of 150 N was applied parallel to the long axis of the implants: to the central fossa of the molar restoration in the single-implant models, and divided equally between the central fossae of the two premolar restorations (75 N each) in the double-implant models. For oblique loading, a total force of 300 N was applied to the lingual inclines of the buccal cusps at a 45° angle to the long axis of the implants: to the molar restoration in the single-implant models, and divided equally between the two premolar restorations (150 N each) in the double-implant models (Figure 1). In all models, loads were distributed over a defined area of the loaded surface rather than applied as concentrated point loads at a single node, avoiding local stress singularities at the point of load application. A total of eight linear static analyses were performed, corresponding to the four models under two loading conditions. Because the oblique load (300 N) was twice the vertical load (150 N), the oblique-versus-vertical comparisons reported in Section 3 and Section 4 reflect the combined effect of a doubled force magnitude and a changed loading direction rather than loading direction alone; since the analyses were linear elastic, the direction-specific component of this effect was isolated by normalizing the oblique-loading stresses to the vertical load magnitude, and both the raw and normalized comparisons are reported. The selected magnitudes (150 N vertical; 300 N oblique at 45°) fall within the range commonly used in comparable single- and posterior-implant FEA studies, where vertical loads of 100–300 N and oblique loads of 100–300 N at 30–45° have been applied [27,39]; moreover, oblique loading is known to increase the maximum stress and strain in bone and implant compared with axial loading because of the added bending moment it introduces [40].

Stress distributions were evaluated separately in the implant components and surrounding bone. Maximum von Mises stress was used to evaluate the implant body, abutment, and screw, whereas maximum (tensile) and minimum (compressive) principal stresses were used for cortical and trabecular bone. Stress concentrations were additionally examined at the crestal, middle, and apical regions of the implant body; the implant–abutment connection; the neck, connection, and body regions of the screw; and the crestal cortical and adjacent trabecular bone. For each double-implant configuration, the configuration-level stress for a given structure was defined as the higher of the two per-site (premolar and molar) values, i.e., the stress at the most heavily stressed site; the per-implant-load interpretation of these values, in which each implant of a double-implant configuration carries half of the applied load, is given in Section 4.

All materials were assumed to be homogeneous, isotropic, and linearly elastic [41,42,43]. The Young’s modulus and Poisson’s ratio assigned to each material are presented in Table 1. Because each model produced a single deterministic value for every evaluated variable, no statistical analysis was performed and the results were compared descriptively.

Values were adopted from previously published finite element studies [41,42,43]. The tooth was modeled as a single material, without separate representation of enamel and dentin, using a Young’s modulus representative of values commonly reported for dentin in comparable implant FEA studies. Although the implant body, abutment, and screw are manufactured from different titanium alloys in reality (e.g., commercially pure titanium for the implant body versus Ti-6Al-4V for the abutment and screw), a single representative Young’s modulus (110 GPa) was used for all titanium components for modeling simplicity.

## 3. Results

Across all configurations, oblique loading produced substantially higher stress values than vertical loading. Numerical results for implant components and peri-implant bone are summarized in Table 2 and in Table 3 and Table 4, respectively. Because the oblique load was twice the vertical load, the raw oblique-to-vertical stress ratios (4.3- to 18.0-fold) overstate the effect of loading direction; normalized per unit load, the increase was 4.0–8.5-fold for the implant body, 3.8–9.0-fold for the abutment, 3.8–6.0-fold for the screw, 2.7–6.5-fold for cortical bone, and 2.2–2.3-fold for trabecular bone (peak values at each site).

### 3.1. Implant Components

**Figure 2:**
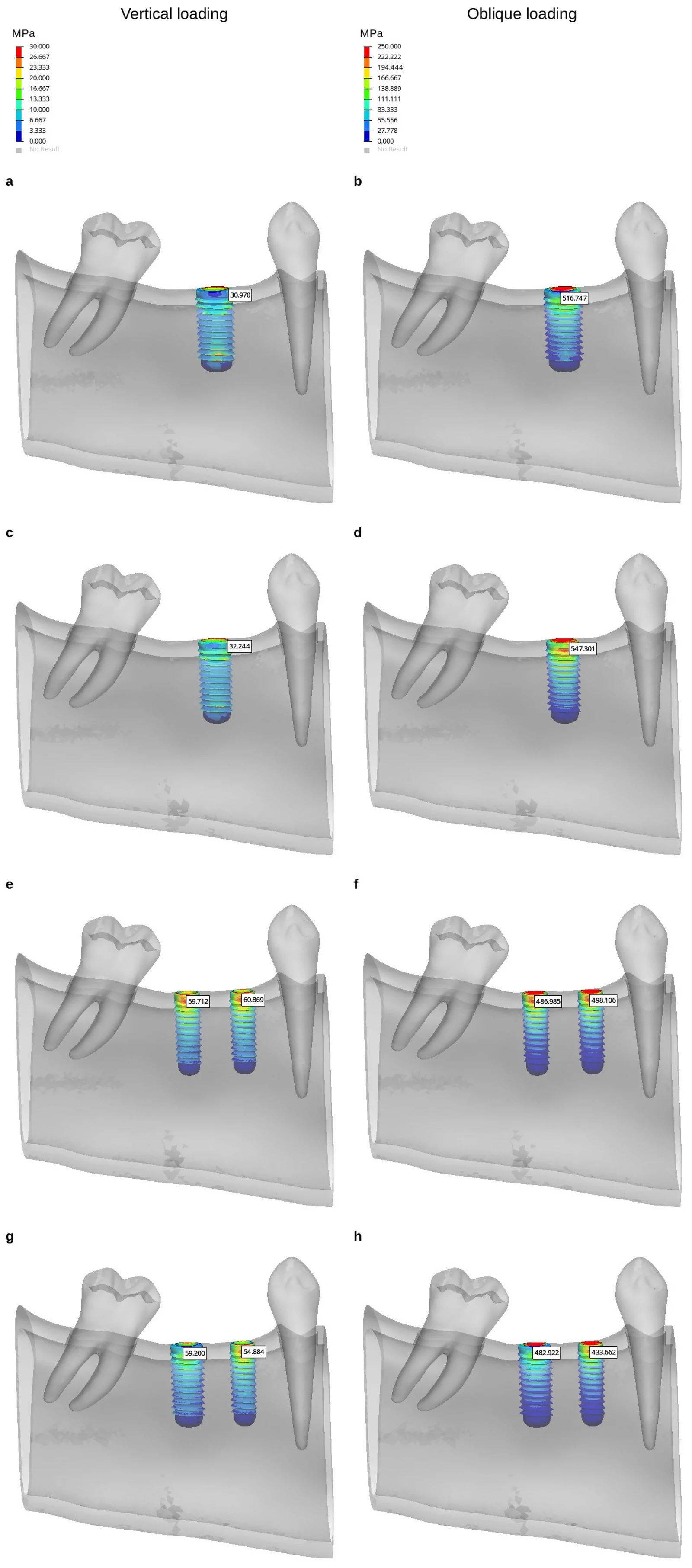
Implant body stress distributions. Panels (**a**–**h**) represent M1 vertical, M1 oblique, M2 vertical, M2 oblique, M3 vertical, M3 oblique, M4 vertical, and M4 oblique conditions, respectively. Stress values are in MPa; the color scale differs between the vertical and oblique loading columns.

**Figure 3:**
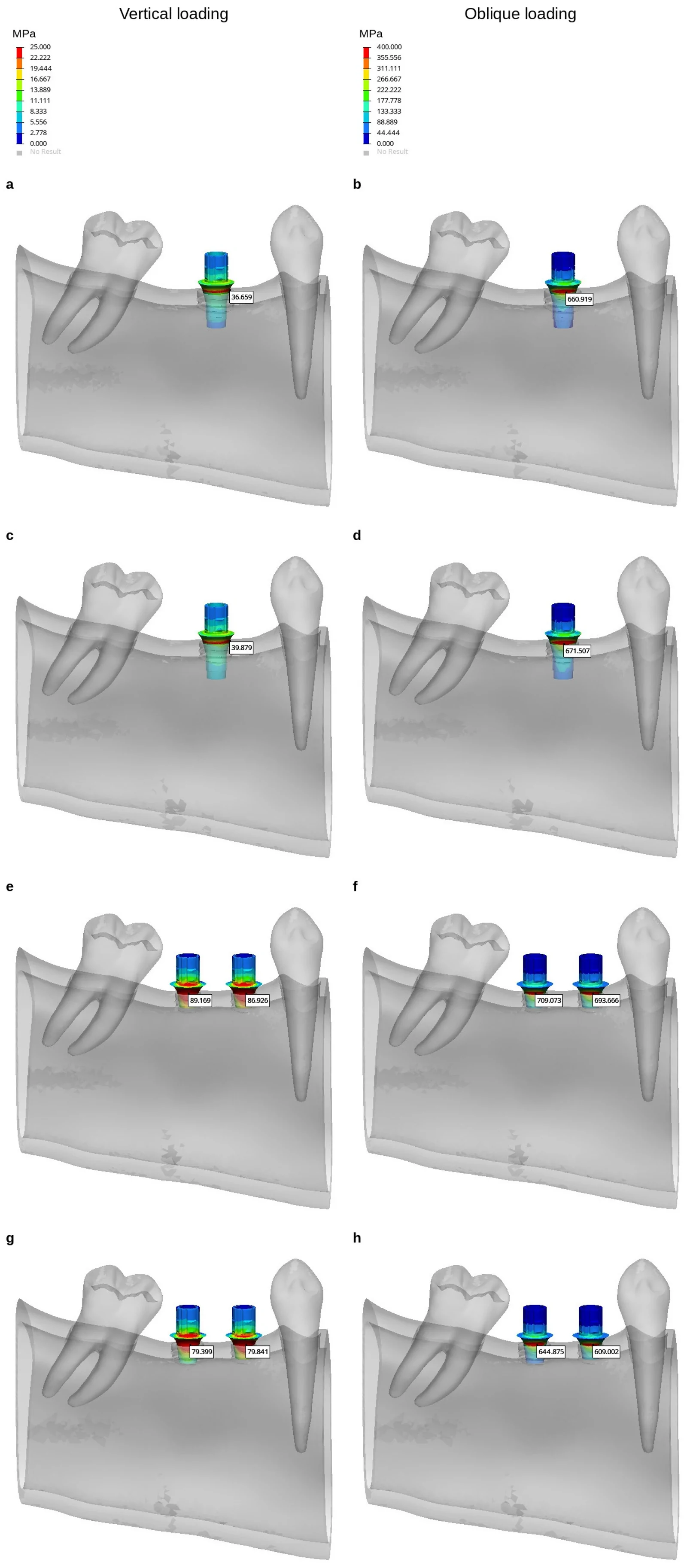
Abutment stress distributions. Panels (**a**–**h**) represent M1 vertical, M1 oblique, M2 vertical, M2 oblique, M3 vertical, M3 oblique, M4 vertical, and M4 oblique conditions, respectively. Stress values are in MPa; the color scale differs between the vertical and oblique loading columns.

**Figure 4:**
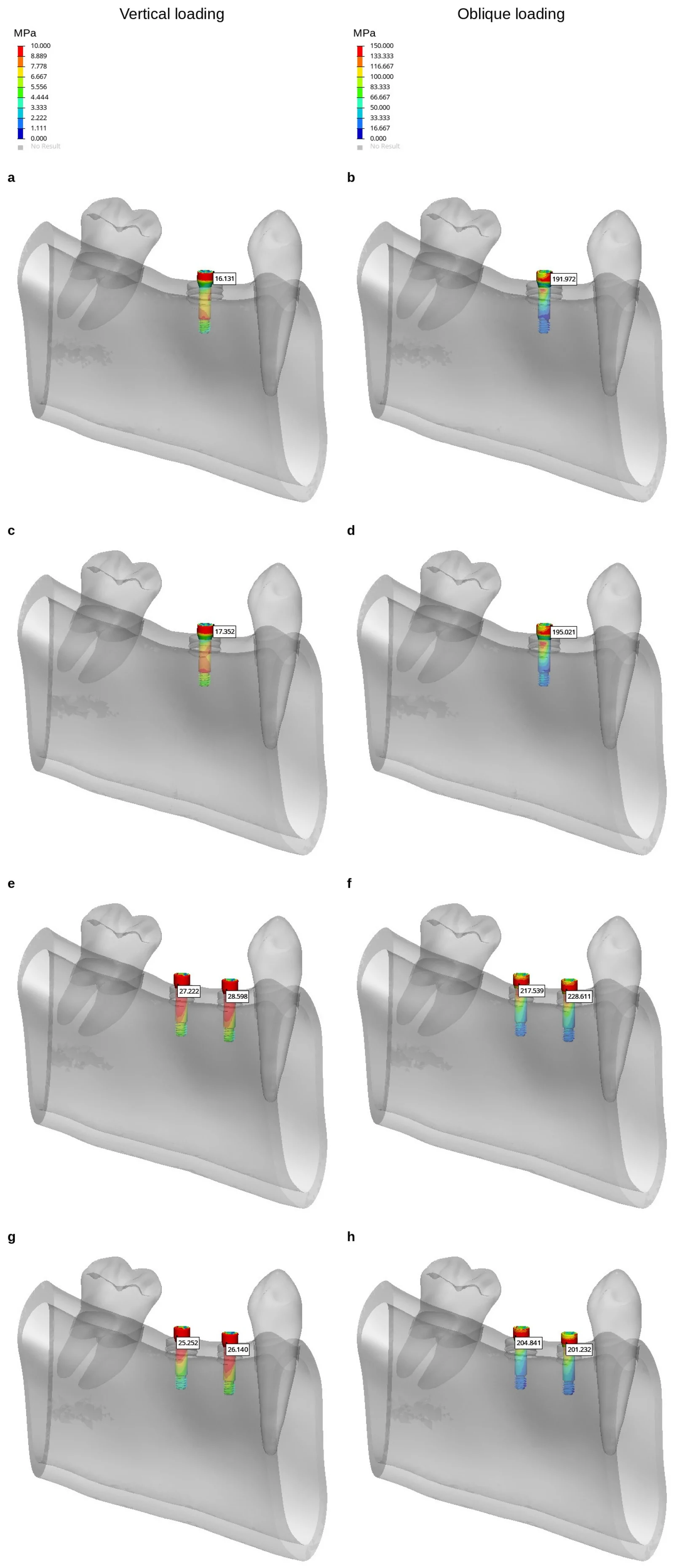
Screw stress distributions. Panels (**a**–**h**) represent M1 vertical, M1 oblique, M2 vertical, M2 oblique, M3 vertical, M3 oblique, M4 vertical, and M4 oblique conditions, respectively. Stress values are in MPa; the color scale differs between the vertical and oblique loading columns.

Under vertical loading, the single-implant configurations (M1 and M2) showed lower implant-body, abutment, and screw stresses than the double-implant configurations, with the lowest values consistently observed in M1 (31.0, 36.7, and 16.1 MPa, respectively). Among the double-implant configurations, M4 generally showed lower component stresses than M3 (Table 2). Comparing the single-implant models, M1 produced 3.9–8.1% lower component stresses than M2 under vertical loading and 1.6–5.6% lower stresses under oblique loading. Comparing the double-implant models, M4 produced 5.8–12.9% lower oblique implant-body, abutment, and screw stresses than M3, with the exception of the M4 molar implant body, where the difference from M3 was negligible (0.8%). The corresponding implant-body, abutment, and screw stress distributions are shown in Figure 2, Figure 3 and Figure 4.

Under oblique loading, implant-body stress was highest in M2 (547.3 MPa) and lowest at the premolar implant of M4 (433.7 MPa) (Table 2; Figure 2). Abutment stress was highest at the molar site of M3 (709.1 MPa) and lowest at the premolar site of M4 (609.0 MPa) (Table 2; Figure 3). Screw stress was lowest in M1 (192.0 MPa) and highest at the premolar site of M3 (228.6 MPa) (Table 2; Figure 4). The contour maps showed that stress concentrations were predominantly localized around the implant neck and the implant–abutment/screw connection regions (Figure 2, Figure 3 and Figure 4).

### 3.2. Peri-Implant Bone

**Figure 5:**
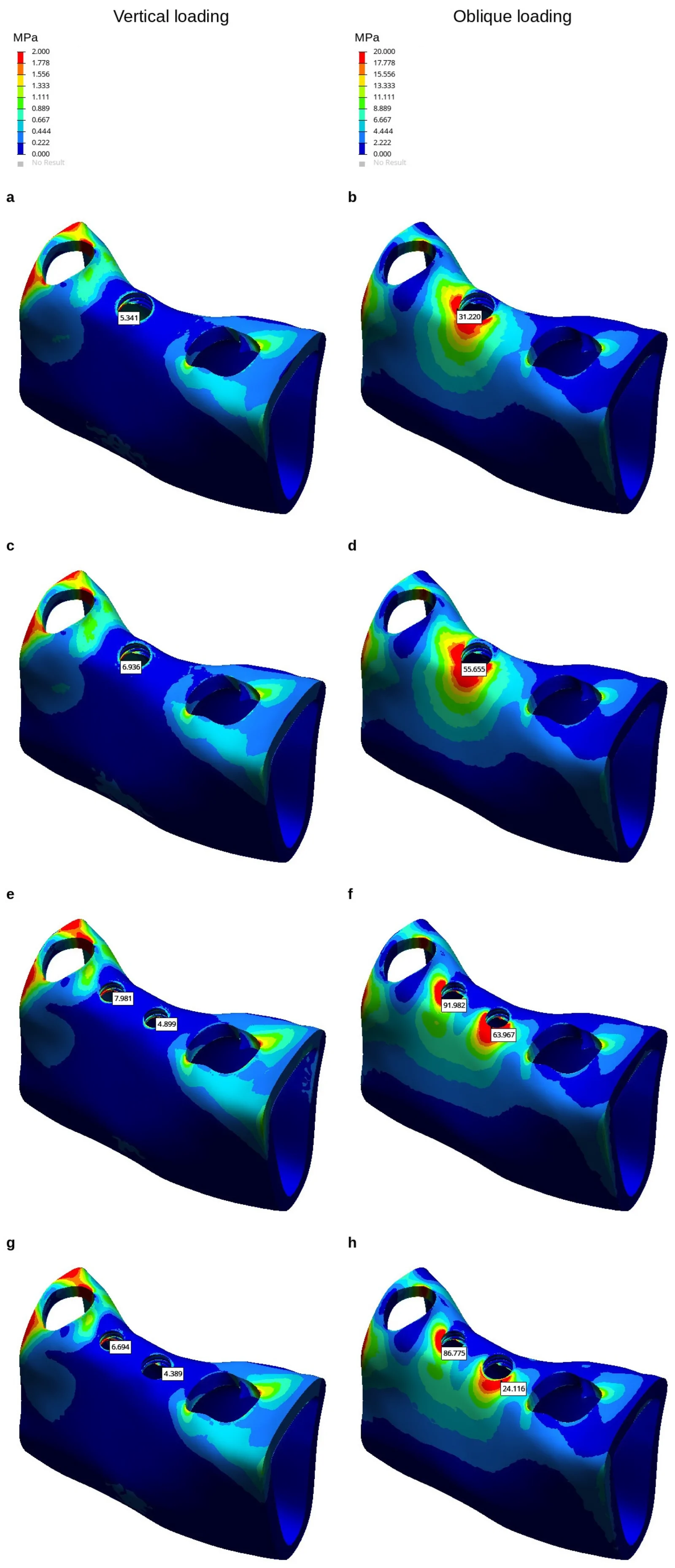
Cortical bone maximum principal (tensile) stress distributions. Panels (**a**–**h**) represent M1 vertical, M1 oblique, M2 vertical, M2 oblique, M3 vertical, M3 oblique, M4 vertical, and M4 oblique conditions, respectively. Stress values are in MPa; the color scale differs between the vertical and oblique loading columns.

For cortical bone, the lowest vertical stress occurred at the molar site of M4 (4.4 MPa), whereas the highest occurred at the premolar site of M3 (8.0 MPa). Under oblique loading, cortical stress was lowest at the molar site of M4 (24.1 MPa) and highest at the premolar site of M3 (92.0 MPa); however, the premolar cortical stress in M4 remained high (86.8 MPa), indicating a marked site-dependent response (Table 3). The corresponding maximum principal stress distributions are shown in Figure 5.

**Figure 6:**
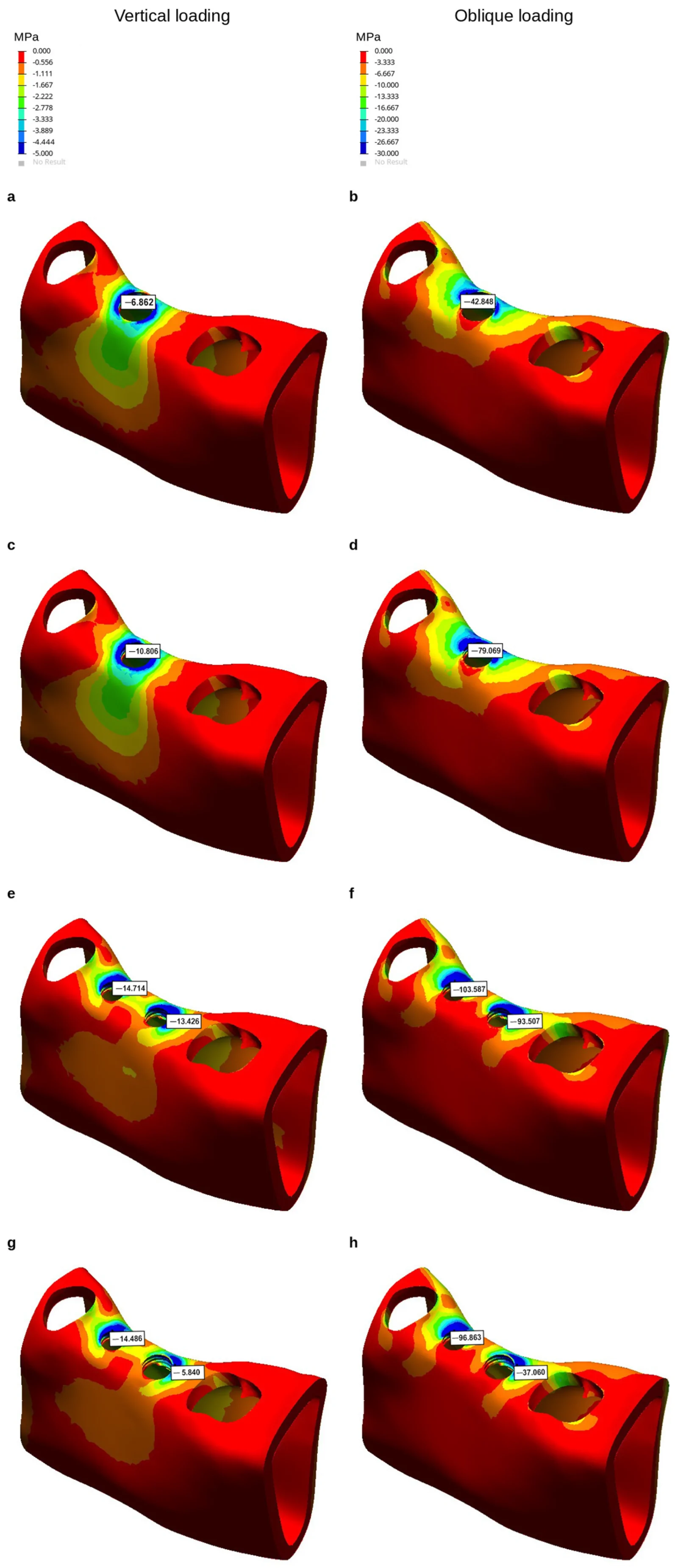
Cortical bone minimum principal (compressive) stress distributions. Panels (**a**–**h**) represent M1 vertical, M1 oblique, M2 vertical, M2 oblique, M3 vertical, M3 oblique, M4 vertical, and M4 oblique conditions, respectively. Negative values indicate compression, and the color scale is reversed relative to Figure 5 and Figure 7 (red, 0 MPa; blue, highest compressive stress). Stress values are in MPa; the color scale differs between the vertical and oblique loading columns.

**Figure 7:**
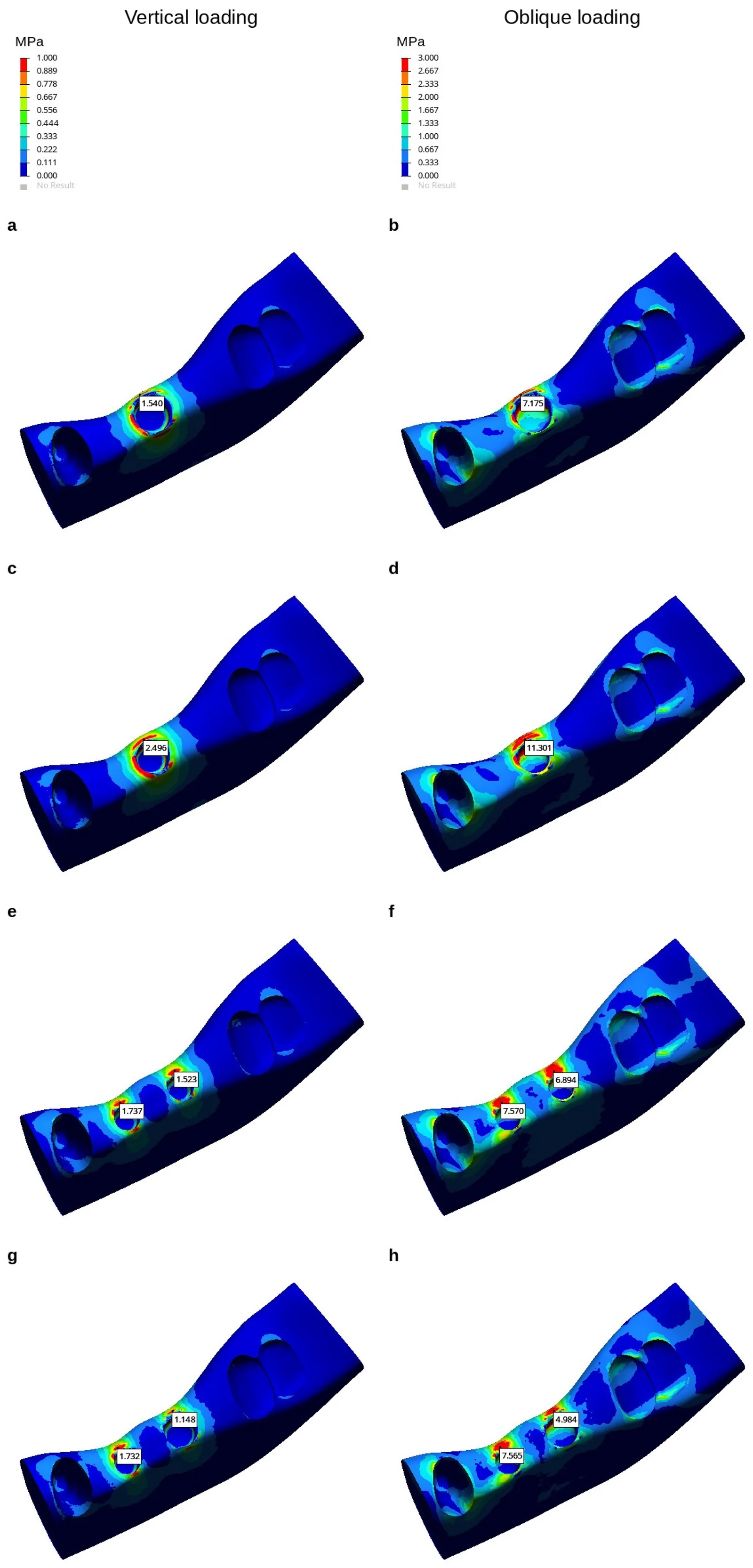
Trabecular bone maximum principal (tensile) stress distributions. Panels (**a**–**h**) represent M1 vertical, M1 oblique, M2 vertical, M2 oblique, M3 vertical, M3 oblique, M4 vertical, and M4 oblique conditions, respectively. Stress values are in MPa; the color scale differs between the vertical and oblique loading columns.

**Figure 8:**
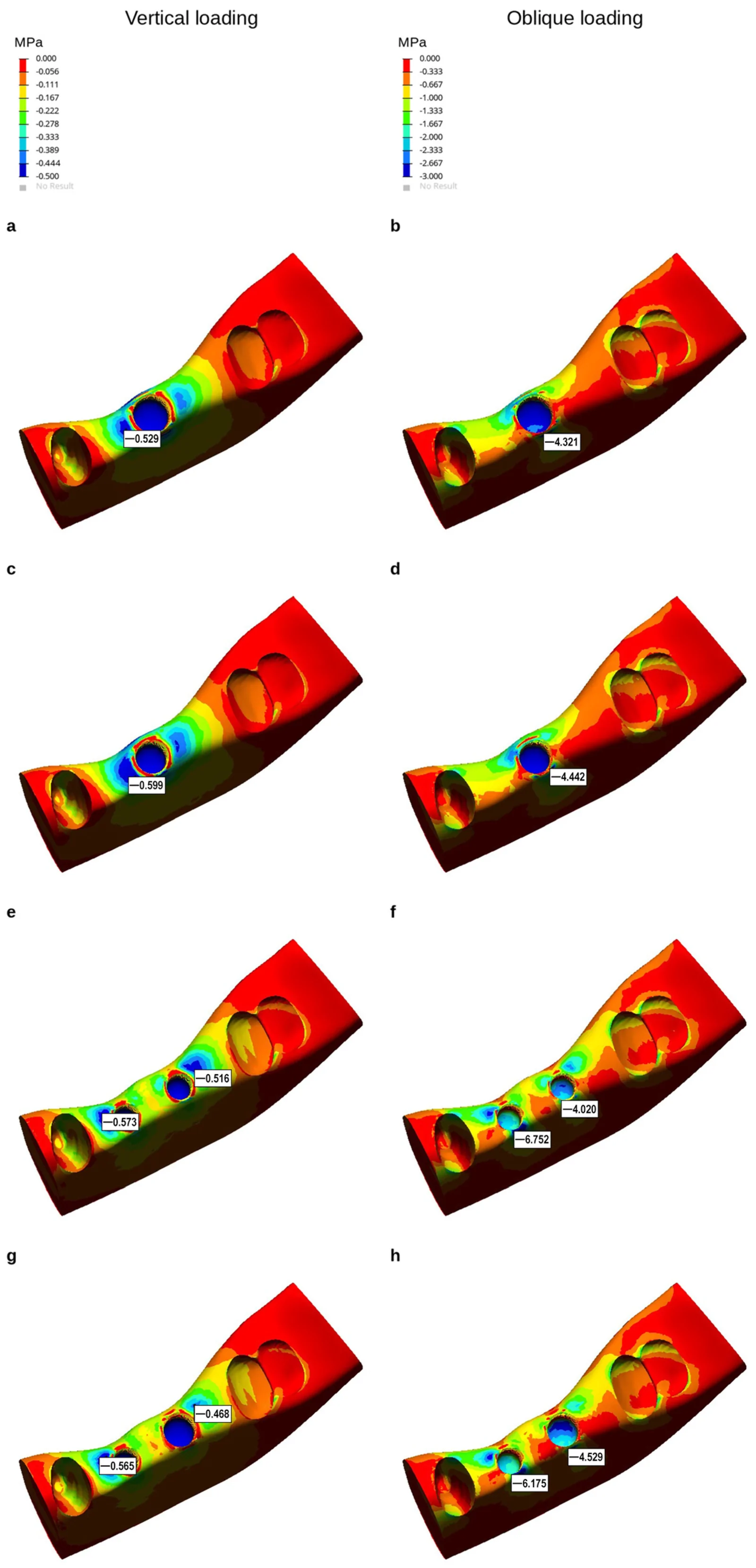
Trabecular bone minimum principal (compressive) stress distributions. Panels (**a**–**h**) represent M1 vertical, M1 oblique, M2 vertical, M2 oblique, M3 vertical, M3 oblique, M4 vertical, and M4 oblique conditions, respectively. Negative values indicate compression, and the color scale is reversed relative to Figure 5 and Figure 7 (red, 0 MPa; blue, highest compressive stress). Stress values are in MPa; the color scale differs between the vertical and oblique loading columns.

Trabecular bone stresses were lower than cortical bone stresses in all configurations. The lowest vertical and oblique trabecular stresses occurred at the molar site of M4 (1.1 and 5.0 MPa, respectively), whereas the highest values occurred in M2 (2.5 and 11.3 MPa, respectively) (Table 3). M1 produced 23.0% and 43.9% lower cortical stress than M2 under vertical and oblique loading, respectively, and 38.4% and 36.5% lower trabecular stress. Within the double-implant configurations, M4 produced cortical and trabecular stresses that were 5.7% and 0.1% lower than M3 at the premolar site under oblique loading, but 62.3% and 27.7% lower at the molar site, reflecting the pronounced site-dependent asymmetry addressed in Section 4. The contour maps showed that bone stress concentrations were predominantly localized in the crestal peri-implant region (Figure 5, Figure 6, Figure 7 and Figure 8), with the trabecular bone distributions presented in Figure 7 and Figure 8.

Minimum principal (compressive) stresses are reported in Table 4 and shown in Figure 6 and Figure 8. In cortical bone, compressive stresses exceeded tensile stresses at all twelve evaluated sites, ranging from −5.8 MPa (M4 molar site, vertical loading) to −103.6 MPa (M3 premolar site, oblique loading). Under oblique loading, the lowest cortical compressive stress occurred in M1 (−42.8 MPa), followed by M2 (−79.1 MPa), whereas the highest values occurred at the premolar sites of M3 (−103.6 MPa) and M4 (−96.9 MPa); at the molar sites, M4 showed lower compressive stress than M3 (−37.1 vs. −93.5 MPa). In trabecular bone, in contrast, tensile stresses exceeded compressive stresses at all twelve sites; compressive stresses ranged from −0.5 to −6.8 MPa and, under oblique loading, were similar for M1 and M2 (−4.3 and −4.4 MPa) and highest at the premolar sites of M3 (−6.8 MPa) and M4 (−6.2 MPa) (Table 4; Figure 8).

## 4. Discussion

This FEA demonstrated component- and site-specific effects of implant diameter, implant number, and loading condition (direction and magnitude) in a narrowed mandibular molar space. Oblique loading increased stress in all evaluated structures. Increasing the diameter of the single implant from 4.1 to 4.8 mm reduced component and peri-implant bone stresses, while replacing the 3.3-mm molar implant with a 4.1-mm implant generally reduced stresses within the double-implant configurations. However, no configuration minimized stress in all structures; therefore, the null hypothesis was rejected.

Among the single-implant models, the 4.8-mm implant produced lower implant-body, abutment, screw, cortical, and trabecular bone stresses than the 4.1-mm implant under both loading conditions. This finding is consistent with previous reports indicating that increasing the implant diameter enlarges the load-transfer area and may reduce stress concentration [10,44]. Nevertheless, the response differed among implant components. Under oblique loading, the highest implant-body stress occurred in the 4.1-mm single-implant model, whereas the highest abutment and screw stresses occurred at different sites in the 3.3–3.3-mm double-implant model. Stress concentration around the implant–abutment connection was consistent with previous biomechanical analyses identifying this region as mechanically susceptible, particularly under nonaxial loading [24,44,45].

Within the double-implant models, replacing the 3.3-mm molar implant with a 4.1-mm implant reduced implant-body, abutment, and screw stresses. However, increasing the number of implants did not uniformly decrease mechanical demand. Under oblique loading, the mixed-diameter configuration showed lower implant-body and abutment stresses than the single-implant configurations, whereas screw stresses remained higher in both double-implant models. These findings suggest that two-implant configurations redistribute rather than uniformly reduce stress. Importantly, screw stress values should be interpreted in relation to implant platform dimensions: the 3.3-mm implant has a narrower internal connection than the 4.1- and 4.8-mm implants and, compared with the single-implant models, a smaller abutment screw (ref. 025.2926 vs. 025.4900); however, the 4.1-mm implant of M4 used the same screw as the 3.3-mm implants. Therefore, similar numerical stress values may not represent equivalent mechanical safety margins. This component-dependent behavior may partly explain conflicting reports favoring either double-implant configurations for load distribution [9,17,18,19] or single implants for peri-implant strain [16]. Clinically, narrow-diameter implants restoring fixed prostheses in the posterior region show survival rates of 92.7% to 100% across studies, with complications mainly involving screw loosening, fractures, and restoration detachment [13], which supports the relevance of component-level evaluation.

Peri-implant bone stresses also demonstrated a marked site-dependent pattern. The 4.8-mm single implant generated lower cortical and trabecular bone stresses than the 4.1-mm single implant under both loading conditions. Within the double-implant configurations, M4 reduced bone stress relative to M3; however, this benefit was not uniformly distributed between implant sites. The 4.1-mm molar implant in M4 showed the lowest individual cortical and trabecular bone stresses, whereas relatively high cortical stress persisted around the 3.3-mm premolar implant under oblique loading. Accordingly, when the higher site-specific value within each configuration was considered, M1 provided the lowest configuration-level bone stress under oblique loading. These findings emphasize that a favorable response around one implant should not be interpreted as favorable biomechanical behavior of the entire configuration and further support the influence of implant diameter on load transfer [10,44]. The magnitude of this mesial–distal asymmetry is itself noteworthy: in M3, the two implants were identical and received identical loads, yet oblique cortical stress was 92.0 MPa at the premolar site versus 64.0 MPa at the molar site, and a larger asymmetry (86.8 vs. 24.1 MPa) was observed in M4. This site-dependent response may reflect local variations in the geometry of the remodeled ridge and the proximity of each implant to the adjacent teeth, in particular the mesially tipped second molar, rather than any difference between the implants themselves or between the two premolar-shaped units of the restoration; these explanations were not tested separately in the present model and should be regarded as hypotheses. This finding also bears directly on the single-versus-double implant comparison: because each implant in a double-implant configuration carries only half of the applied load, a per-implant stress that is not correspondingly lower than the value observed for the equivalent single-implant configuration represents a relative biomechanical disadvantage rather than an advantage, and the comparisons above should be read with this per-implant-load framing in mind. Regarding the direction of these findings, the lower configuration-level bone stress of the single 4.8-mm implant (M1) relative to both double-implant configurations agrees with the finite element study of Mozaffari et al., who reported lower maximum bone stress around a single 4.8-mm implant than around two 3.3-mm implants (3-mm inter-implant and 1.5-mm implant–tooth gaps in a 12.6-mm space) [30], a design similar to M1 and M3 in the present study, and with the lower peri-implant strain reported for single implants by de Carvalho et al. [16]. In contrast, Desai et al. reported well-controlled micromotion for both a 6-mm single wide implant and double implants, with lower von Mises stress for the double implants [14]; differences in implant diameter, loading, and outcome variables (micromotion and von Mises stress rather than component- and site-specific principal stresses) may explain this divergence, and the present results indicate that the conclusion depends on the structure and outcome examined.

Oblique loading produced substantially greater stresses than vertical loading, with stress concentrations predominantly located around the implant neck and connection regions. This pattern is consistent with previous studies identifying the cervical region as a mechanically sensitive load-transfer zone and demonstrating increased bending moments under nonaxial loading [22,24,46]. Although FEA-derived stress values cannot directly predict clinical failure, increased mechanical demand in these regions may be relevant to biological and technical complications such as marginal bone changes, screw loosening, prosthetic fracture, and implant fracture [21,47,48,49]. From a biomechanical perspective, the present findings therefore support minimizing nonaxial loading and considering implant diameter, number, anatomical position, and individual component behavior together when selecting an implant configuration for a narrowed molar space.

The clinical interpretation of these values also warrants caution regarding their absolute magnitude. Under oblique loading, implant-body stresses of 433.7–547.3 MPa and abutment stresses of 609.0–709.1 MPa approached or exceeded the reported yield strength of commercially pure grade 4 titanium (approximately 480–550 MPa) and approached that of titanium alloys such as Ti-6Al-4V (approximately 800–900 MPa); cortical bone stresses of 86.8–92.0 MPa at the premolar sites of M3 and M4 approached commonly cited estimates of the ultimate tensile strength of cortical bone and exceeded thresholds associated with pathological overload, as described by Frost [20]. These peak values may partly reflect the idealized boundary conditions of the model—bonded contacts, sharp geometric transitions, and nodal load application—which are known to generate local stress concentrations in FEA, rather than purely representing the mechanical demand that would occur clinically; they should therefore be interpreted as comparative indicators among configurations rather than as absolute predictors of material failure.

Because minimum principal (compressive) stresses were evaluated in addition to maximum principal (tensile) stresses (Table 4; Figure 6 and Figure 8), the two stress types could be compared directly. In cortical bone, compressive stresses exceeded tensile stresses at all twelve evaluated sites (e.g., −103.6 vs. 92.0 MPa at the M3 premolar site under oblique loading), as expected under off-axis loading, whereas in trabecular bone, tensile stresses exceeded compressive stresses at all sites (e.g., 7.6 vs. −6.8 MPa at the same site). Under oblique loading, the ranking of cortical stresses among configurations was identical for tension and compression (M1 < M2 < M4 < M3), and the difference between the two implant sites of M3 was pronounced for tension (92.0 vs. 64.0 MPa) but small for compression (−103.6 vs. −93.5 MPa), which may indicate that the two implants transferred load similarly and that the tensile difference is related to local bone geometry rather than to unequal load sharing.

Compared with the reported strength of human compact bone (approximately 135 MPa in tension and 205 MPa in compression along the longitudinal axis, and approximately 53 and 131 MPa, respectively, in the transverse direction [50]), the peak cortical tensile stresses (86.8–92.0 MPa) corresponded to about 64–68% of the longitudinal tensile strength and exceeded the transverse tensile strength, whereas the peak compressive stress (−103.6 MPa) corresponded to about 51% of the longitudinal and 79% of the transverse compressive strength. Thus, although compressive stresses were larger in magnitude, they were farther from the reported strength limits than tensile stresses, which therefore appear to be the more limiting criterion in cortical bone. Comparing peak first and third principal stresses with separate tensile and compressive strengths of cortical bone has been used previously for cortical bone around miniscrew implants [51]. These comparisons are indicative only, because the reference strengths were obtained from non-mandibular bone and peak stresses in a linear model with bonded interfaces sensitive to local geometry and mesh. For trabecular bone, the relative magnitude of tensile and compressive strength is not well established (higher tensile than compressive strength has been reported for cancellous bone [52]); therefore, no strength-based threshold was applied to the trabecular values.

This study has several limitations inherent to FEA. The modeled materials were assumed to be homogeneous, isotropic, and linearly elastic, which does not fully reproduce the heterogeneous and anisotropic behavior of biological tissues. Complete osseointegration was assumed at the bone–implant interface, and individual variations in bone quality and occlusal conditions were not considered. Furthermore, bonded contact was assumed between all components, and screw preload was not incorporated into the models. Because screw stress is one of the study’s primary outcomes, this simplification should be regarded as a substantive limitation rather than a general FEA caveat: with bonded, non-preloaded interfaces, the implant–abutment–screw assembly behaves mechanically as a single body, and the reported screw-stress values reflect a shared deformation rather than the mechanics of a preloaded, frictional joint. Accordingly, the screw-stress comparisons reported here should be interpreted as comparative indicators of relative mechanical demand among configurations rather than as predictors of screw loosening or fracture risk; models incorporating a preloaded screw and frictional implant–abutment and screw contact are needed to evaluate these outcomes directly. A single standardized anatomical model was used, without variability in cortical bone thickness or bone quality, and occlusal contacts were represented in a simplified form; these factors may influence the absolute stress magnitudes and the generalizability of the present findings to the range of clinical presentations of a narrowed molar space. In the single-implant models, the crown filled the entire mesiodistal space and therefore overhung the implant platform; this geometry, which reflects a restoration that fills the entire narrowed space on a narrow implant, may increase bending moments under oblique loading and was not varied in the present study. Because the finite element models were constructed and are held by a third-party commercial provider under a confidentiality agreement that restricts their disclosure or reuse, a dedicated model/mesh/boundary-condition figure could not be newly generated for this manuscript. Static loading was applied; therefore, dynamic and repetitive masticatory forces and fatigue-related effects were not evaluated. Accordingly, the present findings should be interpreted as comparative biomechanical tendencies rather than direct predictors of clinical performance. Future studies incorporating different bone qualities, dynamic and fatigue loading, alternative implant–abutment connection conditions, and clinical validation are warranted.

## 5. Conclusions

Within the limitations of this FEA, implant diameter, implant number, anatomical site, and loading condition (direction and magnitude) influenced stress distribution in a narrowed mandibular molar space, and no single configuration minimized stress in all evaluated structures. The 4.8-mm single-implant configuration showed a more favorable response for screw and configuration-level peri-implant bone stresses, whereas the mixed-diameter double-implant configuration reduced implant-body and abutment stresses under oblique loading but did not uniformly reduce screw or peri-implant bone stresses. Because screw preload was not modeled, the reported screw-stress comparisons should be interpreted as relative mechanical demand among configurations rather than as direct predictors of screw loosening or fracture risk. These findings support a component- and site-specific approach to implant selection rather than identification of a universally superior configuration.

## Figures and Tables

**Table 1:** Mechanical properties assigned to the materials used in the finite element models.

Materials		
Material	Young’s Modulus (GPa)	Poisson’s Ratio
Cortical bone	13.7	0.30
Trabecular bone	1.37	0.30
Titanium (implant, abutment and screw)	110	0.30
Monolithic zirconia superstructure	220	0.30
Cement layer	8.3	0.35
Periodontal ligament	0.05	0.45
Tooth (enamel and dentin, single material)	18.6	0.31

**Table 2:** Maximum von Mises stress (MPa) in implant components under vertical and oblique loading.

Configuration/Site	Vertical			Oblique		
	Implant Body	Abutment	Screw	Implant Body	Abutment	Screw
M1—4.8 mm single	31.0	36.7	16.1	516.7	660.9	192.0
M2—4.1 mm single	32.2	39.9	17.4	547.3	671.5	195.0
M3—3.3 mm premolar	60.9	86.9	28.6	498.1	693.7	228.6
M3—3.3 mm molar	59.7	89.2	27.2	487.0	709.1	217.5
M4—3.3 mm premolar	54.9	79.8	26.1	433.7	609.0	201.2
M4—4.1 mm molar	59.2	79.4	25.3	482.9	644.9	204.8

M1, 4.8 mm single implant; M2, 4.1 mm single implant; M3, 3.3 + 3.3 mm double implant; M4, 4.1 + 3.3 mm double implant.

**Table 3:** Maximum principal stress (MPa) in cortical and trabecular bone under vertical and oblique loading.

Configuration/Site	Vertical		Oblique	
	Cortical	Trabecular	Cortical	Trabecular
M1—4.8 mm single	5.3	1.5	31.2	7.2
M2—4.1 mm single	6.9	2.5	55.7	11.3
M3—3.3 mm premolar	8.0	1.7	92.0	7.6
M3—3.3 mm molar	4.9	1.5	64.0	6.9
M4—3.3 mm premolar	6.7	1.7	86.8	7.6
M4—4.1 mm molar	4.4	1.1	24.1	5.0

M1, 4.8 mm single implant; M2, 4.1 mm single implant; M3, 3.3 + 3.3 mm double implant; M4, 4.1 + 3.3 mm double implant.

**Table 4:** Minimum principal stress (MPa; negative values indicate compression) in cortical and trabecular bone under vertical and oblique loading.

Configuration/Site	Vertical		Oblique	
	Cortical	Trabecular	Cortical	Trabecular
M1—4.8 mm single	−6.9	−0.5	−42.8	−4.3
M2—4.1 mm single	−10.8	−0.6	−79.1	−4.4
M3—3.3 mm premolar	−14.7	−0.6	−103.6	−6.8
M3—3.3 mm molar	−13.4	−0.5	−93.5	−4.0
M4—3.3 mm premolar	−14.5	−0.6	−96.9	−6.2
M4—4.1 mm molar	−5.8	−0.5	−37.1	−4.5

M1, 4.8 mm single implant; M2, 4.1 mm single implant; M3, 3.3 + 3.3 mm double implant; M4, 4.1 + 3.3 mm double implant.

## Data Availability

The numerical results generated in this study are presented in the article. The CAD/finite element model files belong to the commercial partner that performed the analyses and are kept confidential to prevent their reuse in other studies; they are therefore not publicly available. Further inquiries can be directed to the corresponding author.

## Supplementary Materials

The following supporting information can be downloaded at https://www.mdpi.com/article/10.3390/jfb17100533/s1. Table S1: Number of elements and nodes of each component in the four finite element models; Table S2: Mesh convergence data for the implant body (maximum von Mises stress) and the peri-implant bone (maximum principal stress).
