# Supplementary material for: Single Versus Double Implants in a Narrowed Mandibular Molar Space: A Finite Element Analysis
Source: J Funct Biomater. 2026 Oct 21;17(10):533. doi: 10.3390/jfb17100533 (PMC13705665; doi:10.3390/jfb17100533)
Supplement: Supplementary file 1 [file jfb-17-00533-s001.zip › jfb-4593894-supplementary.pdf]

Supplementary Materials

Single versus Double Implants in a Narrowed Mandibular Molar Space: A Finite Element Analysis

Table S1. Number of elements and nodes of each component in the four finite element models (M1, 4.8 × 10 mm single implant; M2, 4.1 × 10 mm single implant; M3, 3.3 + 3.3 mm double implants; M4, 4.1 + 3.3 mm double implants). Implant 1 is the single implant (M1, M2) or the premolar-site implant (M3, M4); implant 2 is the molar-site implant (M3, M4). The totals correspond to the values given in the Methods.

**Component**	**M1**		**M2**		**M3**		**M4**	
	**Elements**	**Nodes**	**Elements**	**Nodes**	**Elements**	**Nodes**	**Elements**	**Nodes**
First premolar (adjacent tooth)	16,636	4,402	16,636	4,402	16,636	4,402	16,636	4,402
Second molar (adjacent tooth)	50,216	12,713	50,216	12,713	50,216	12,713	50,216	12,713
Periodontal ligament, first premolar	98,003	28,490	98,003	28,490	98,003	28,490	98,003	28,490
Periodontal ligament, second molar	177,410	50,552	177,410	50,552	177,410	50,552	177,410	50,552
Zirconia restoration (single molar crown in M1/M2; splinted premolar-form restoration in M3/M4)	140,155	33,866	140,155	33,866	308,625	74,158	308,625	74,158
Cement layer, implant 1 (single implant in M1/M2; premolar-site implant in M3/M4)	35,786	12,055	35,786	12,055	35,724	12,037	35,749	12,044
Cement layer, implant 2 (molar-site implant in M3/M4)	–	–	–	–	35,697	12,031	35,735	12,042
Ti-base abutment, implant 1	99,908	25,549	99,908	25,549	62,861	16,945	62,861	16,945
Ti-base abutment, implant 2	–	–	–	–	62,946	16,958	72,670	19,006
Abutment screw, implant 1	34,339	9,045	34,339	9,045	34,339	9,045	34,339	9,045
Abutment screw, implant 2	–	–	–	–	34,165	9,011	34,165	9,011
Implant body, implant 1 (M1: 4.8 × 10 mm; M2: 4.1 × 10 mm; M3/M4: 3.3 × 10 mm)	183,933	46,139	151,962	38,475	109,763	28,028	109,763	28,028
Implant body, implant 2 (M3: 3.3 × 10 mm; M4: 4.1 × 10 mm)	–	–	–	–	109,723	28,011	151,670	38,005
Cortical bone	236,083	61,231	212,255	54,765	261,551	67,053	333,695	84,205
Trabecular bone	525,734	125,761	441,989	105,460	532,365	126,402	586,466	139,610
**Total**	**1,598,203**	**409,803**	**1,458,659**	**375,372**	**1,930,024**	**495,836**	**2,108,003**	**538,256**

Table S2. Mesh convergence test on the M1 model under oblique loading. The listed sizes are local mesh sizes applied in the critical regions, such as the areas around the implant holes, and not global element sizes. Relative error (%) = [(value of updated mesh − value of previous mesh) / value of updated mesh] × 100. The 0.1-mm local mesh size (relative error < 3% for both criteria) was used in these regions for all final analyses.

Local mesh size (mm)	Implant body, maximum von Mises stress (MPa)	Relative error (%)	Peri-implant bone, maximum principal stress (MPa)	Relative error (%)
0.4	424.5	–	25.5	–
0.3	473.2	10.29	28.7	11.26
0.2	505.3	6.35	30.5	5.93
0.1	516.7	2.21	31.2	2.29
